# Characterization of Polysaccharides Extracted from Pulps and Seeds of *Crataegus azarolus* L. var. *aronia*: Preliminary Structure, Antioxidant, Antibacterial, *α*-Amylase, and Acetylcholinesterase Inhibition Properties

**DOI:** 10.1155/2020/1903056

**Published:** 2020-05-29

**Authors:** Ilhem Rjeibi, Rihab Zaabi, Warda Jouida

**Affiliations:** ^1^Research Unit of Macromolecular Biochemistry and Genetics, Faculty of Sciences of Gafsa, 2112 Gafsa, Tunisia; ^2^Faculty of Sciences of Gabés, University of Gabés, 6072, Tunisia

## Abstract

Polysaccharides from the pulps (CAP) and seeds (CAS) of *Crataegus azarolus* L. var. *aronia* were extracted by hot water method. Both polysaccharides were characterized by scanning electron microscopy (SEM), Congo red test, FT-IR spectroscopy, and their antioxidant, *α*-amylase, antiacetylcholinesterase, and antibacterial activities were evaluated. CAP showed the highest total carbohydrate (82.35%) and uronic acid (29.39%) contents. The Congo red test revealed the lack of triple-helical conformation for both polysaccharides. The comparison of both infrared spectra indicated similar patterns with the presence of typical bands of polysaccharides. However, the microstructure of both samples indicated differences when analyzed by SEM. CAP displayed higher antioxidant, *α*-amylase, and acetylcholinesterase inhibitory activities. Besides, CAP showed the strongest antimicrobial effects against seven microorganisms and, notably, the Gram-positive bacteria. Overall, the results suggest that polysaccharides from *C. azarolus* L. var. *aronia* may be considered as novel sources of antioxidants and recommended as enzyme inhibitory agents in food and pharmaceutical industries.

## 1. Introduction

Polysaccharides are biomacromolecules widely distributed in algae, plants, animals, and microorganisms. Plant polysaccharides have proved to be potential sources of natural antibacterial, antioxidants, immunomodulatory, antitumor, hepato-cardioprotective, and neuroprotective compounds [1–3]. They have been increasingly applied because they are sourced naturally, and they impart less toxicity, biodegradability, and fewer side effects than synthetic ones. Polysaccharides are also widely used as emulsifiers, gelling agents, thickeners, and fat replacers in functional food, cosmetics industries, and biological medicine, including drug delivery and tissue engineering [4]. Over the past decade, there has been a wave of studies into finding new sources of polysaccharides that could hotspot a potential technological interest over existing commercial polysaccharides.

The genus *Crataegus* spp., which belongs to the Rosaceae, is largely distributed in Africa, North Europe, and North America [5]. This genus is commonly known as hawthorn in English and Zaarour in Arabic. The fruits of *Crataegus* spp. are commonly eaten as edible food. In addition, fruits, leaves, and flowers have long been used as a traditional medicine to cure various diseases such as asthma, insomnia, flu, coughs, and bronchitis, and headache, respiratory, and cardiovascular problems [6, 7]. Previous research has shown that hawthorn exerts a variety of pharmacological effects, including antioxidant, antidiabetic, antimicrobial, antiviral, anti-inflammatory, antithrombotic, antihyperlipidemic, cardioactive, hepatoprotective, and hypotensive activities [8]. Numerous biochemical studies have demonstrated that hawthorn is a valuable source of bioactive components (e.g., minerals, sugar alcohols, phenolic acids, essential oil, organic acids, tannins, vitamin, flavonoids, and polysaccharides) [8, 9]. Polysaccharides and oligosaccharides extracted from the fruits and flowers of *Crataegus* spp. possess various human health-promoting effects, such as anticoagulant (for *C. monogyna*) [10] and hypolipidemic activities (for *C. pinnatifida*) [11]. Likewise, several reports have demonstrated the antioxidant and probiotic properties of polysaccharides extracted from *C. pinnatifida* [12, 13].

Among plant species *Crataegus azarolus* L. var. *aronia* (Yellow Azarole) is native to the Mediterranean countries, which have long been used in Tunisian traditional medicines to prevent cancer, diabetes, sexual weakness, and cardiovascular diseases [14]. Previous studies revealed that the leaves, flowers, and fruits of *C. azarolus* had various biological activities including antimicrobial, antioxidant, antihyperglycemic, and antihyperlipidemic activities [15, 16]. These potential health benefits are related to their high content in many natural active compounds, such as flavonoids, minerals, sugar alcohols, carotenoids, polyphenols, amino acids, and tannins [14, 17].

However, to the best of our knowledge, none of the previous studies have focused on the extraction of polysaccharides from *C. azarolus* L. var. *aronia* and the evaluation of their antioxidant, antibacterial, *α*-amylase, and acetylcholinesterase inhibition properties. In this study, two polysaccharides from *C. azarolus* were extracted and structurally characterized preliminarily. Then, their biological activities *in vitro* were evaluated.

## 2. Materials and Methods

### 2.1. Plant Material

Fresh fruits of *Crataegus azarolus* L. var. *aronia* were collected from Gafsa (Northwestern Tunisia, 36° 46′ 34^″^ N latitude and 8° 41′ 05^″^ E longitude) between October and November 2018. The plant was identified by Professor Elkadri Lefi, at the Department of Biology, Faculty of Sciences of Gafsa, Tunisia. A voucher specimen (MSE 0795) was deposited at the herbarium in the Faculty of Sciences Gafsa, Tunisia. The pulps and seeds of the fruits were separated, dried, and crushed individually to obtain a fine powder.

### 2.2. Extraction of CAS and CAP

The powdered pulps and seeds (60 g, each) were defatted with 95% ethanol and petroleum ether with continuous stirring for 24 h. The residues were dried and then extracted with hot water at 90°C for 5 h (three-time, 3 × 5 h). Following centrifugation at 4500 rpm for 10 min, the supernatants were mixed with 95% cold ethanol (3: 1, *v*/*v*) at 4°C overnight. Precipitates were dissolved in distilled water and deproteinized using Sevag reagent (chloroform/butanol 4: 1, *v*/*v*). The deproteinized mixture was dialyzed for 3 days (with 3500 Da cut-off, *Spectra/Por™*, Fisher Scientific, Illkirch, France) and lyophilized to obtain the water-soluble polysaccharides from *C. azarolus* seeds and pulps named, respectively, CAS and CAP.

Finally, the CAP and CAS extraction yields were calculated.

### 2.3. Characterization of CAS and CAP

#### 2.3.1. Chemical Composition

Total carbohydrates were assessed using the phenol–sulfuric acid method [18], and concentrations were determined against the glucose standard. The total neutral sugar, total phenolic compounds, and uronic acid contents were estimated using, respectively, the sulfuric resorcinol method [19], Folin-Ciocalteu method [20], and *m*-hydroxydiphenyl test [21]. The protein content was determined using the Bradford method [22], and concentrations were estimated against the bovine serum albumin standard. Ash content was determined according to AOAC methods [23].

#### 2.3.2. Infrared Spectroscopic Analysis (FT-IR)

CAP and CAS were individually mixed with potassium bromide powder and pressed into pellets. The spectra were analyzed using Fourier transform infrared spectrophotometer (Shimadzu, FT-IR-8400S spectrophotometer equipped with IR solution version 1.10) in the range of 400–4000 cm^−1^.

#### 2.3.3. Scanning Electron Microscopy

CAP and CAS were examined by scanning electron microscopy (SEM) model JEOL (JSM-IT100). Each dried polysaccharide was mounted on a metal stub and was sputtered with gold. The images were observed at different magnifications (35x and 250x).

#### 2.3.4. Helix-Coil Transition Analysis

The conformational structure of CAP and CAS was analyzed using the Congo red assay [24]. In brief, the two polysaccharides (2 mg/mL each) were individually mixed with 2 mL of 100 *μ*M Congo red solution. Different volumes of NaOH solution (2 M) were added to the mixture to achieve a final concentration of 0-0.5 M. Meanwhile, the solution prepared without adding polysaccharides was considered as the control. The maximum UV-vis absorption was measured from 250 to 550 nm using Analytik Jena spectrophotometer.

### 2.4. Antioxidant Activity

#### 2.4.1. DPPH Radical Scavenging Activity

The scavenging capacity of CAP and CAS against DPPH radical was assayed using the method of Bersuder et al. [25]. Aliquots of polysaccharides (500 *μ*L) at different concentrations (0.1-4 mg/mL) were mixed with DPPH solution (125 *μ*L, 0.2 mM) and deionized water (375 *μ*L), then incubated for 1 h in the dark. The positive standards (butylated hydroxytoluene and vitamin C) were prepared using the same procedure. The absorbance was measured at 517 nm. The scavenging activity of DPPH radicals was calculated according to

(1) $$\begin{array}{c} \text{Scavenging activity }\left(\%\right)=\frac{\text{Ab}{\text{s}}_{\text{Control}}-\text{Ab}{\text{s}}_{\text{Sample}}}{\text{Ab}{\text{s}}_{\text{Control}}}\times 100. \end{array}$$

#### 2.4.2. H_2_O_2_ Scavenging Activity

The scavenging capacity of CAP and CAS against H_2_O_2_ radical was conducted according to the modified procedure of Liu et al. [26]. Briefly, 0.5 mL of CAP and CAS at various concentrations (0.1, 0.5, 1.5, 2.5, 3, and 4 mg/mL) were individually mixed with 0.1 M phosphate buffer (1.2 mL, pH 7.4) and 40 mM H_2_O_2_ solution (0.3 mL), then incubated for 10 min at room temperature. The positive standard in this assay was vitamin C. The absorbance of each sample was measured at 230 nm. The scavenging activity of H_2_O_2_ radicals was calculated according to Equation (1).

#### 2.4.3. Fe^2+^-Chelating Activity

The chelating capacity of ferrous ions by CAP and CAS was assessed using ferrozine reagent procedure as described previously with slight modifications [27]. Solution of polysaccharides (500 *μ*L) at different concentrations (0.1-4 mg/mL) were mixed with 2 mM FeCl_2_ (100 *μ*L), 5 mM ferrozine (200 *μ*L), and deionized water (200 *μ*L) and incubated for 10 min at 25°C. In the control solution, the sample was replaced by deionized water. The positive standard in this assay was EDTA (ethylenediaminetetraacetic acid). The ferrous ion chelation activity was calculated according to Equation (1).

#### 2.4.4. Lipid Peroxidation Inhibition Activity

The inhibition effect of CAP and CAS on lipid peroxidation was carried out as described by Yen and Hsieh [28] using mice liver homogenate as the lipid-rich media, FeCl_2_–H_2_O_2_ as inducer, and ascorbic acid as the standard. The livers of Swiss albinos mice obtained from the departmental animal house at the Faculty of Sciences Gafsa were dissected, washed, and homogenized into ice-cold Tris-HCl buffer (1%, pH 7.4). The resulting reaction mixture was centrifuged for 30 min at 9000 rpm at 4°C. Aliquots (500 *μ*L) were mixed with solutions of polysaccharides (500 *μ*L) at different concentrations (0.5-6 mg/mL), then 50 *μ*L of FeCl_2_ (0.5 mmol/L) and H_2_O_2_ (0.5 mmol/L) was added to start lipid peroxidation. After incubation for 30 min at 37°C, the trichloroacetic acid (500 *μ*L, 20%) was added to precipitate proteins, and the mixture was centrifuged. The thiobarbituric acid (1 mL, 0.8%) was added to the obtained supernatant, then heated in boiling water for 9 min. The absorbance of the supernatant was recorded at 532 nm. The inhibition was calculated using

(2) $$\begin{array}{c} \text{Inhibition rate }\left(\%\right)=\frac{1-\left(\text{A}1-\text{A}2\right)}{\text{A}0}\times 100, \end{array}$$

where A0 and A1 were, respectively, the absorbance without and with the test sample, and A2 was the absorbance without liver homogenate.

### 2.5. Enzyme Inhibitory Activity Assays

#### 2.5.1. Acetylcholinesterase Inhibition

The antiacetylcholinesterase effects of CAP and CAS on AChE was analyzed using the modified procedure of Ellman et al. [29]. Briefly, a mixture of 300 *μ*L (50 mM) Tris-HCl buffer pH 8, 100 *μ*L of each polysaccharide, and 30 *μ*L AChE solutions was well shaken and incubated for 15 min. Then, 130 *μ*L of AChI (acetylthiocholine iodide) and 440 *μ*L of (3 mM) DTNB (5,5′-Dithiobis-(2-nitrobenzoic acid)) were added. Galantamine was used as the positive control. The absorbance was measured at 412 nm.

The inhibition activity was calculated using

(3) $$\begin{array}{c} \text{Inhibition activity }\left(\%\right)=\frac{1-\text{ES}}{E}\times 100. \end{array}$$

ES and *E* were the respective activity of enzyme with and without the test sample.

#### 2.5.2. *α*-Amylase Inhibition

The *α*-amylase inhibitory activity of CAP and CAS was assessed as described by Oboh et al. [30] with minor modifications. Firstly, 500 *μ*L of different concentrations (0.1-5 mg/mL) of each polysaccharide prepared in PBS (20 mM) were mixed with *α*-amylase (500 *μ*L, 1.0 U/mL) prepared in NaCl (6.0 mM) and incubated for 10 min at 37°C. Then, potato starch solutions (500 *μ*L, 1%) was added to the mixture and re-incubated for 10 min at 37°C. Finally, the reaction was stopped using 1 mL of dinitrosalicylic acid (DNS) reagent and heated in boiling water for 5 min. The absorbance of the resulting mixture was measured at 520 nm. The acarbose was used as the positive control.

The inhibitory activity was estimated as follows:

(4) $$\begin{array}{c} \text{Inhibition }\left(\%\right)=\frac{\text{Ab}{\text{s}}_{520}\text{control}-\text{Ab}{\text{s}}_{520\;}\text{sample}}{\text{Ab}{\text{s}}_{\text{A}1}\text{control}}\times 100. \end{array}$$

### 2.6. Antibacterial Activity

#### 2.6.1. Microorganisms

Microorganisms used in this study represent pathogenic species commonly associated with sanitary relevance. These bacterial organisms, including Gram-positive and Gram-negative, are the main source that causes severe infections in humans. Antibacterial activity of polysaccharides was tested against Gram-positive and Gram-negative bacterial strains from the American Type Culture Collection. The test organisms used here are as follows: *Escherichia coli* (ATCC 35218), *Enterococcus faecalis* (ATCC 29212), *Pseudomonas aeruginosa* (ATCC 27853), *Listeria monocytogenes* (ATCC 19117), *Klebsiella pneumoniae* (ATCC 13883), *Staphylococcus aureus* (ATCC 25923), *Bacillus cereus* (ATCC 11778), and *Salmonella typhimurium* (ATCC 23564).

#### 2.6.2. Disc Diffusion Assay

Antibacterial activity of CAP and CAS (15 mg/mL) was performed by disc diffusion method. The suspensions of bacteria (200 *μ*L, 10^6^ CFU/mL, with CFU/mL of bacterial cells estimated by absorbance at 600 nm) were spread on Mueller–Hinton (MH) agar (Sigma-Aldrich) already cast into Petri dishes. Next, impregnated sterile paper discs (6 mm diameter, 1 mm thickness) with 10 *μ*L of each polysaccharide were deposited individually on Petri dishes, then incubated for 24 h at 37°C. The antimicrobial activity was evaluated by measuring the inhibition zone surrounding the discs (mm) using vernier caliper (accuracy 0.02 mm). Gentamicin at 20 *μ*g/disc was used as the positive control.

#### 2.6.3. Minimum Inhibitory Concentration (MIC)

The MIC of different polysaccharides was performed using the 96-well microdilution method as previously described by Gullon et al. [31]. The microorganism suspension was prepared in order to obtain a final cell density of about 10^6^ CFU/mL. Serial dilutions of each polysaccharide from 1.56 to 25 mg/mL were prepared using MH broth. Subsequently, 100 *μ*L of the diluted samples were distributed into the microplate. To the above dilutions, equal volumes (100 *μ*L) of the different bacterial suspensions (10^6^ CFU/mL) were added. Plates were then incubated at 37°C for 24 h. The MIC was considered as the lowest concentration of drugs or substances able to inhibit any visible microbial growth.

### 2.7. Statistical Analysis

Statistical analysis was performed using the SPSS version 18.0 software. All data were analyzed using a one-way analysis of variance (ANOVA) (Tukey test). All values are expressed as mean ± standard deviation (SD) and *p* < 0.05 considered significant.

## 3. Results and Discussion

### 3.1. Extraction Yields and Physicochemical Property of Polysaccharides

The extraction yields and chemical composition of the crude polysaccharides CAS and CAP obtained from *C. azarolus* seeds and pulps, respectively, are summarized in Table 1. The extraction yield of CAP (6.92%) was higher than that of CAS (2.58%). Pawlaczyk-Graja [10] reported that the extraction yield of polysaccharides from *C. monogyna* flowers and fruits ranged from 16.7 to 4.1%. Our results were in line with previous reports suggesting that the differences in species, conditions, and type of extraction procedure could influence the extraction yield of polysaccharides [32]. CAP and CAS showed relative high ash content (3.08% in CAP and 3.99% in CAS). This high content could be related to the presence of residual inorganic salt after the purification of polysaccharides. Protein contents were 0.83% and 5.68% for CAP and CAS, respectively. CAP showed 82.35% of carbohydrate and 52.86% of neutral sugar contents, which were higher than those of CAS (64.93% of carbohydrate and 45.25% of neutral sugar). Uronic acid contents were 29.49% for CAP and 19.68% for CAS suggesting the presence of acidic polysaccharides [33].

### 3.2. FT-IR Spectrometric Analysis

**Figure 1:**
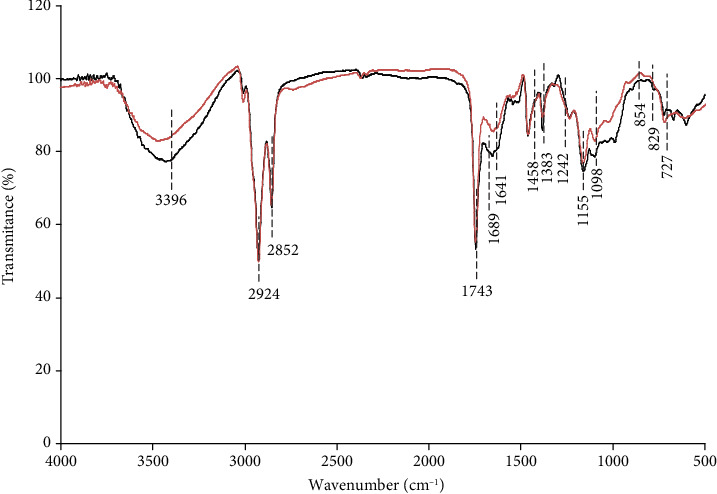
Fourier-transform infrared spectroscopy spectra of CAS and CAP.

For quick evaluation of the important functional groups and linkage of polysaccharides, the FT-IR spectrum of CAP and CAS was performed, and results are illustrated in Figure 1. The comparison of both spectra indicated similar patterns. Both spectra showed broad peaks at around 3396 cm^−1^ which were ascribed to the stretching vibration of hydroxyl groups in the constituent sugar residues [34]. The peaks at approximately 2924 and 1242 cm^−1^ were related to the C–H asymmetric stretching vibration [35]. The peak located at 1537 cm^−1^ suggested the presence of phenolic groups [36]. The absorbance peaks at about 1743 cm^−1^, 1689 cm^−1^, and 1522 cm^−1^ were attributed to carboxyl and carboxylate vibrations, showing the presence of uronic acids [37, 38], which was verified by chemical analysis. The region at around 1000–1200 cm^−1^ indicates the presence of pyranose [39]. The characteristic absorptions at 854 cm^−1^ and 921 cm^−1^ might be attributed to the existence of *α* and *β* configurations in CAP and CAS [40].

### 3.3. SEM Analysis

**Figure 2:**
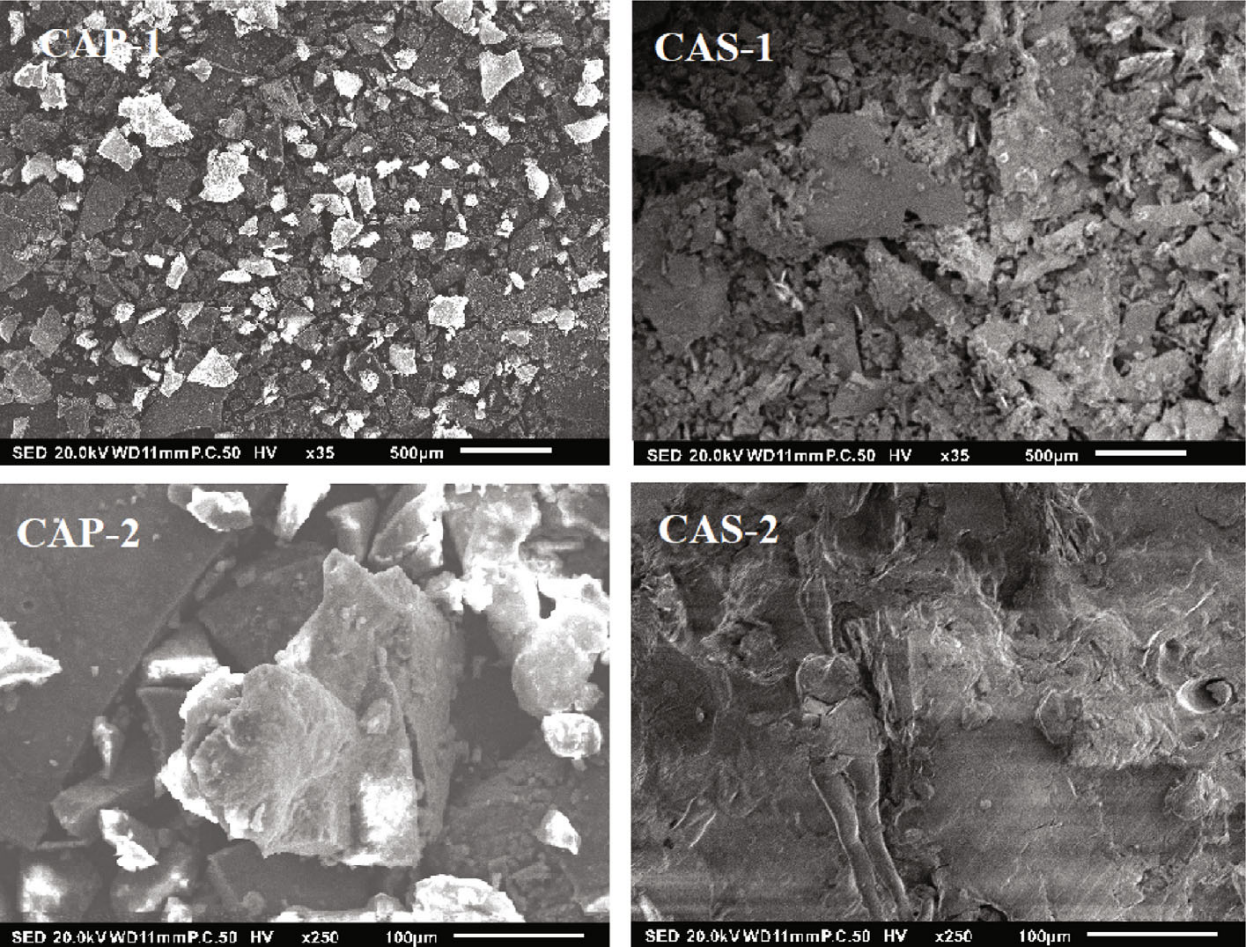
Scanning electron micrographs of polysaccharides from CAP and CAS (1: magnification 35x, scale bar 500 *μ*m; 2: magnification 250x, scale bar 100 *μ*m).

The microstructure of the two samples indicated differences when analyzed by SEM at different magnifications (35x and 250x) (Figure 2). CAS consisted of many small particles in aggregation with irregular shape and dimensions [41], whereas CAP has a relatively uniform surface with schistose substances. Nep and Conway [42] reported that the method of the preparation of the plant material may affect the shape and surface topology of polysaccharides.

### 3.4. Triple-Helical Conformation Analysis

**Figure 3:**
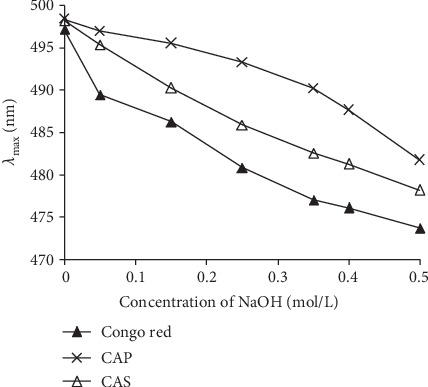
Changes in absorption wavelength maximum (*λ*max) of CAS and CAP Congo red complex at various NaOH concentrations. The maximum absorption wavelength of the mixture was determined by ultraviolet scanning (250-550 nm).

The Congo red is a sensitive technique to confirm the conformational structure of the polysaccharides as a triple-helical structure [43]. In general, when a polysaccharide with a triple-helical conformation is mixed with Congo red, the *λ*max will shift towards a longer wavelength [33]. But this *λ*max will decrease rapidly with the increase of NaOH concentrations (Figure 3). Our results showed that the *λ*max of Congo red-CAP complex and Congo red-CAS complex presented a comparable shift trend as Congo red alone once the concentration of NaOH increased from 0 to 0.5 mol/L, suggesting no triple-helical conformation for both polysaccharides.

### 3.5. *In Vitro* Antioxidant Activities Analysis

#### 3.5.1. DPPH Radical Scavenging Activity

**Figure 4:**
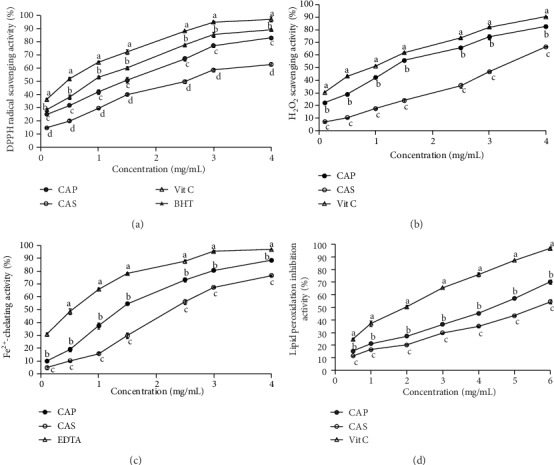
Antioxidant activities of CAP and CAS with different methods. (a) DPPH scavenging activity; (b) H_2_O_2_ scavenging activity; (c) Fe^2+^-chelating activity; (d) lipid peroxidation inhibition activity. Positive controls consisted of Vit C, BHT, and EDTA (vitamin C, butylated hydroxytoluene, and ethylenediaminetetraacetic acid, respectively). Each value is expressed as mean ± SD of three replicates. Different letters represent the significant difference (*p* < 0.05) at the same concentration for different samples.

The scavenging rates of DPPH radicals by both polysaccharides increased with the increase of concentrations ranging from 0.1 to 4 mg/mL (Figure 4(a)). The scavenging effects attained maximum values of 83.2% and 63.74% for CAP and CAS, respectively, at the concentration of 4 mg/mL. It can be seen that the scavenging capacity of *C. azarolus* polysaccharides to DPPH radicals was similar to other polysaccharides. For example, polysaccharides extracted from *Crataegus pinnatifida* Bunge (CPPu) have been reported to have a DPPH radical scavenging activity of 87.4% at a concentration of 5 mg/mL [44]. CAS had lower efficacy to scavenge DPPH (SC_50_ = 2.56 mg/mL) than CAP (SC_50_ = 1.47 mg/mL). However, the free radical scavenging ability of CAP and CAS was lower than that of the positive control (vitamin C, SC_50_ = 0.47 mg/mL). The DPPH radical scavenging ability of CAP and CAS was higher than that of polysaccharide extracted from the fruits of *Morus nigra* [45] but lower than that from *Nitrari retusa* fruit polysaccharide [3]. Previous studies suggested that the content of uronic acid is a key factor in the antioxidant effects of polysaccharides [46, 47]. Our study showed that CAP and CAS contained 29.49 and 19.68% uronic acid, respectively, which might serve as an important factor in the antioxidant capacity of both polysaccharides. Moreover, Blois [48] has demonstrated that hydroxyl groups are involved in the higher DPPH radical scavenging ability, which was consistent to our study.

#### 3.5.2. H_2_O_2_ Scavenging Activity

H_2_O_2_ scavenging effect of CAP and CAS are illustrated in Figure 4(b). Clearly, there was a dose-dependent relationship between each polysaccharide concentrations and the antioxidant activity. The scavenging efficiencies of CAP, CAS, and vitamin C at 4 mg/mL were 82.48%, 66.51%, and 90.25%, respectively. The SC_50_ value of the CAP was 1.34 mg/mL, which was 2.39-fold lower than that of CAS, indicating that CAP is a more potent radical scavenger than CAS. In the literature, there are no studies on the H_2_O_2_ scavenging effect of the *Crataegus* species presented in the present study. In comparison with polysaccharides extracted from the fruits of other species, the antiradical activity of CAP was found near to that of *Lycium europaeum* (SC_50_ = 1.19 mg/mL) [49] and lower than that of *Nitraria retusa* (SC_50_ = 2.03 mg/mL) [3]. Previous studies reported that the scavenging abilities of polysaccharides might depend on functional groups as COOH and OH present in saccharides structures [50], which was similar to the present study.

#### 3.5.3. Ferrous Ion-Chelating Activity

Metal ions (Cu^2+^, Pb^2+^, and Fe^2+^) are well known to be engaged in the generation of free radicals and indirectly contribute to lipid peroxidation and DNA damage. The Fe^2+^-chelating activity of the CAP and CAS was evaluated, and results are illustrated in Figure 4(c). Both polysaccharides exhibited good concentration-dependent ferrous ion-chelating ability. The chelating potential increased with increasing concentration up to 4 mg/mL and was always stronger for CAP than CAS. This might be due to a stronger chelating ability for CAP compared to CAS. At 4 mg/mL, the chelating potential of CAP, CAS, and EDTA were 88.43%, 76.53%, and 96.76%, respectively. The EC_50_ values of CAP and CAS were 1.38 and 2.28 mg/mL, respectively, which were higher than that of EDTA (0.51 mg/L). In the literature, there are no studies on the Fe^2+^-chelating activity of the *Crataegus* species presented in the present study. The ferrous ion-chelating ability of CAP was found to be close to that of polysaccharides extracted from *Malva aegyptiaca* (EC_50_ = 1.15 mg/mL) [51]. It has been reported that biomolecules including some functional groups like COH can easily chelate ferrous (Fe^2+^) ions. In addition, the substances, which own two or more functional groups of OH, COOH, SH, S, CO, and O, had a structure-function relationship [52]. Accordingly, the chelating activity of CAP and CAS might be in part linked to the presence of the strong Fe^2+^-chelating groups in their structure.

#### 3.5.4. Liver Lipid Peroxidation Inhibition Activity

Lipid peroxidation is widely recognized as a key process in diverse diseases [26]. In the literature, there are no studies on the lipid peroxidation inhibition activity of the *Crataegus* species presented in the present study. The effects of both polysaccharides on FeCl_2_–H_2_O_2_-induced lipid peroxidation in mice liver are illustrated in Figure 4(d). The result showed that liver lipid peroxidation was effectively inhibited by CAS and CAP at all tested concentrations. At 6 mg/mL, the inhibition effects of CAP, CAS, and vitamin C were 70.07%, 54.32%, and 96.57%, respectively. The EC_50_ values of the CAP and CAS were 4.44 and 5.52 mg/mL, respectively, which were lower than that of vitamin C (2.29 mg/mL). It has been documented that the protective effects of natural antioxidants (like polysaccharides) on lipid peroxidation induced by Fe^2+^/H_2_O_2_ system might be assigned to their scavenging abilities on H_2_O_2_ and OH radical [53]. Another report [26] revealed that polysaccharides with high metal ion-chelating activities are able to inhibit peroxidation by interfering with the free radical reaction chains.

### 3.6. Enzyme Inhibitory Activity Assays

#### 3.6.1. Acetylcholinesterase (AChE) Inhibition

**Figure 5:**
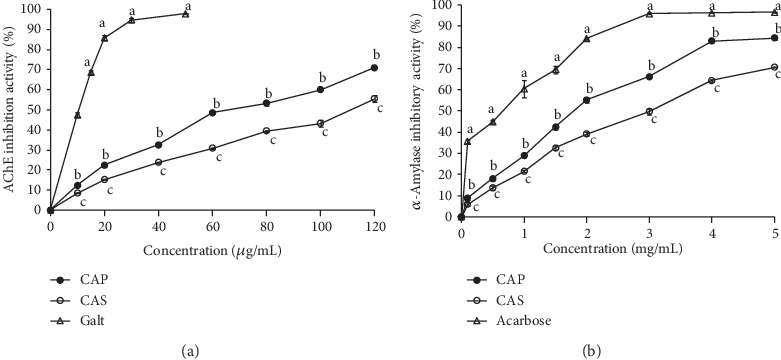
Enzyme inhibitory activities of CAP and CAS with various concentrations. (a) AChE inhibitory activity; (b) *α*-amylase inhibitory activity. Each value is expressed as mean ± SD of three replicates. Different letters represent the significant difference (*p* < 0.05) at the same concentration for different samples.

Alzheimer's disease (AD) is an example of a neurodegenerative disease which affects the memory. One of the goals of AD treatment is to increase the level of acetylcholine in the brain by inhibiting the activity of AChE [54]. Due to the lack of effective treatments for AD and the considerable side effects associated with the use of neuroprotective drugs, researchers are constantly looking for new and more effective therapies from medicinal plants to improve the loss of neuronal cells and brain restoration [55, 56]. Natural antioxidants have been often evinced to have beneficial effects in the prevention of memory impairment. Several investigations have demonstrated the neuroprotective and antioxidant effects of phenolic compounds extracted from hawthorn seeds [57, 58]. These properties were explained by their inhibitory effects on lipid peroxidation and free radicals. Other studies have demonstrated the anticholinesterase activity of phenolic compounds isolated from *C. oxyacantha* [59]. However, the enzyme inhibitory effect of polysaccharides from these plants has not yet been reported. In this study, the effects of both polysaccharides extracted from *C. azarolus* on AChE inhibitory activities are presented in Figure 5(a). The inhibitory effect of CAP and CAS was proportional to the concentration (10-120 *μ*g/mL). The inhibitory effect of CAS was 55.46% at a concentration of 120 *μ*g/mL, which was weaker than the CAP (71.03%) under the same concentration. As summarized in Table 2, CAP displayed important AChE inhibitory activity (EC_50_ = 61.56 *μ*g/mL) as compared to CAS (EC_50_ = 115.94 *μ*g/mL). In this study, the anticholinesterase drug galantamine (an alkaloid isolated from the bulbs and flowers of *Galanthus caucasicus* and FDA-approved drug) was used as a positive control. Results showed that the activity of CAP was less than that of galantamine (EC_50_ = 10.53 *μ*g/mL). AChE inhibitory activity of CAP and CAS were higher than those of *Physalis alkekengi* and *Flammulina velutipes* polysaccharides [60, 61]. This implies that CAS and CAP could be potential inhibitors of AChE and beneficial for human memory. The modulation of the cholinergic system could be one of the pharmacological mechanisms used by *Crataegus* to improve memory problems. In this inhibitory mechanism, polysaccharides (inhibitors) bind to the same active site as the enzyme substrate, and this implies a nonmetabolizable response [62].

#### 3.6.2. *α*-Amylase Inhibition

Among the available procedures, inhibition of *α*-amylase has seemed to be an important therapeutic target for the prevention and management of type 2 diabetes mellitus [63]. When the activity of *α*-amylase is inhibited, the increase of blood glucose concentrations can be delayed. There were a few researches on the inhibition activity of *α*-amylase by *Crataegus* sp. extracts [8]. As far as we know, the present study is the first to describe the *in vitro* antidiabetic effects of polysaccharides from *C. azarolus*. As illustrated in Figure 5(b), both polysaccharides displayed *α*-amylase inhibitory activity in a dose-dependent manner at the range from 0.1 to 5.0 mg/mL. The EC_50_ value of CAP was about 1.81 mg/mL, which was more effective than that of CAS (EC_50_ = 3.01 mg/mL) but less effective than that of acarbose (EC_50_ = 0.82 mg/mL) (Table 2). The inhibitory effects of polysaccharides from *C. azarolus* were similar to that from *Diaphragma juglandis fructus* [64], but also more efficient than that from *Corbicula fluminea* [65] and blackcurrant fruits [66], which have been demonstrated as potent antihyperglycemic agents *in vivo*. Authors have suggested that the high antihyperglycemic capacity of polysaccharides was related to their structure, the configuration of glycosidic bonds, monosaccharide composition, and the high uronic acid content. Other studies have shown that the inhibition mechanism could be that the carboxyl group and the hydroxyl group of polysaccharides could react with the amino acid residues of the digestive enzymes, which caused a reduction in the *α*-amylase activity [67].

### 3.7. Antimicrobial Activity

Antimicrobial activity of CAP and CAS tested against seven microorganisms is summarized in Table 3. The result evidenced that the antibacterial effect of both polysaccharides varied with bacterial species. The inhibition zone diameter of the two polysaccharides against tested bacteria ranged from 10.16 to 16.65 mm and from 8.61 to 11.66 mm for CAP and CAS, respectively. CAP revealed the best antimicrobial effect against *L. monocytogenes* and *B. cereus*. However, CAS displayed the highest inhibition activity toward *E. faecalis*. Moderate antibacterial activity was observed against *K. pneumoniae* with inhibition zones of 10.16 and 9.16 mm for CAP and CAS, respectively. The antimicrobial activity of polysaccharides was also estimated as minimal inhibitory concentration (MIC) (Table 3). Results proved that Gram-positive bacteria were more sensitive to both polysaccharides than Gram-negative. These findings were in accordance with previously published searches [68, 69]. They reported that the outer membrane of Gram (-) bacteria may act as a barrier against hyperacidification, which would result in differences in the resistance of Gram-positive and Gram-negative bacteria to the action of antimicrobial drugs. The mechanisms related to the antimicrobial activity of polysaccharides were still not clear and deserve to be deepened. The broad-spectrum antimicrobial potential of CAP and CAS may be explained by their higher total sugar contents [48]. He et al. [70] reported that the inhibition effect of polysaccharides may be explained by their abilities to induce the disruption of the cell wall of bacteria and to enhance ion permeability leading cell death. Further, DNA might be decomposed into small fragment after the polysaccharide has penetrated into the cell, which can make the bacteria unable to develop resistance. The best activity against *Listeria monocytogenes* was observed with CAP (MIC < 1.56 mg/mL). In the literature, there are no studies on the antimicrobial activity of the *Crataegus* species presented in the present study. The results of MICs denoted that MIC values of polysaccharides from *C. azarolus* found in this study were lower than those obtained in previous findings, which were on the order of 6.25 and 25 mg/mL for polysaccharide from *Saussurea controversa* and from 3.12 to 100 mg/mL for polysaccharide from *Lallemantia royleana*, respectively [71, 72].

## 4. Conclusion

In the present study, two polysaccharides (CAP and CAS) were extracted from *C. azarolus* fruits, and their physiochemical properties were characterized using FT-IR, SEM, and Congo red test. Results of FT-IR analysis indicated that CAP and CAS have similar functional groups that are typical of polysaccharides. Both polysaccharides were devoid of helical conformation. CAP had the highest H_2_O_2_ and DPPH radicals scavenging activities and maximum chelating activity on ferrous ion. *In vitro* CAP remarkably decreased liver lipid peroxidation levels induced by FeCl_2_–H_2_O_2_. Both polysaccharides successfully inhibited AChE and *α*-amylase activities and exhibited effective antimicrobial properties against seven pathogenic bacteria. Altogether, our studies suggest that *C. azarolus* fruits can be further used in food production as a useful natural antioxidant ingredient. Nevertheless, additional studies deserve to be carried out which will elucidate a clear structure-activity relationship.

## Figures and Tables

**Table 1:** Global composition of CAP and CAS extracted from *Crataegus azarolus*.

	Yield (%, *w*/*w*)	Carbohydrate (%, *w*/*w*)	Neutral sugar (%, *w*/*w*)	Proteins (%, *w*/*w*)	Uronic acid (%, *w*/*w*)	Polyphenolics (%, *w*/*w*)	Ash (%, *w*/*w*)
CAP	6.92 ± 0.15^b^	82.35 ± 0.23^b^	52.86 ± 0.19^b^	0.83 ± 0.01^b^	29.49 ± 0.04^b^	1.10 ± 0.01^b^	3.03 ± 0.08^a^
CAS	2.58 ± 0.05^a^	64.93 ± 0.41^a^	45.25 ± 0.31^a^	5.68 ± 0.05^a^	19.68 ± 0.10^a^	2.13 ± 0.16^a^	3.99 ±0.04^b^

Values are means ± SD of three separate experiments. Different letters indicate a comparison between the two polysaccharides at a level of *p* < 0.05.

**Table 2:** Inhibition activity (IC_50_ values) of CAS and CAP on studied enzymes.

	CAP	CAS	Galantamine	Acarbose
AChE (IC_50_, *μ*g/mL)	61.56 ± 0.64^b^	115.94 ± 4.68^c^	10.53 ± 0.23^a^	—
*α*-Amylase (IC_50_, mg/mL)	1.81 ± 0.03^b^	3.01 ± 0.08^c^	—	0.82 ±0.04^a^

Values are means ± SD of three separate experiments. Different letters indicate a comparison between the samples at a level of *p* < 0.05.

**Table 3:** The antimicrobial activity and MICs of CAP and CAS.

Microorganism	Diameters of inhibition zone (mm)		MICs (mg/mL)	
	CAP	CAS	CAP	CAS
Gram negative				
*Escherichia coli* (ATCC 35218)	12.48 ± 0.02	11.25 ± 0.05	3.12	6.25
*Klebsiella pneumoniae* (ATCC 13883)	10.16 ± 0.04	9.16 ± 0.07	6.25	12.5
*Salmonella typhimurium* (ATCC 23564)	13.21 ± 0.01	11.62 ± 0.02	1.56	3.12
Gram positive				
*Bacillus cereus* (ATCC 11778)	14.49 ± 0.01	8.61 ± 0.02	1.56	3.12
*Listeria monocytogenes* (ATCC 19117)	16.65 ± 0.05	11.4 ± 0.11	<1.56	1.56
*Staphylococcus aureus* (ATCC 25923)	12.36 ± 0.04	10.15 ± 0.05	3.12	6.25
*Enterococcus faecalis* (ATCC 29212)	13.95 ± 0.05	11.66 ± 0.04	3.12	1.56

MIC: minimum inhibitory concentration. Values are means ± SD of three separate experiments.

## Data Availability

All data included in this study are available upon request by contacting the corresponding author.
